# Patterns of proximal interphalangeal joint dislocations

**DOI:** 10.1177/17531934251405707

**Published:** 2026-01-20

**Authors:** Charles Bewsey, Grey Giddins

**Affiliations:** 1 Exeter Medical School, University of Exeter, UK; 2 Department of Orthopaedic Surgery, Royal United Hospital, UK

**Keywords:** Finger dislocation, hand trauma, injury pattern, open dislocation, PIPJ, proximal interphalangeal joint dislocation

## Abstract

**Introduction:**

The aim of the study was to understand finger proximal interphalangeal joint (PIPJ) dislocations better by reviewing the demographics and biomechanics of a cohort of PIPJ dislocations.

**Methods:**

All cases of PIPJ dislocations presenting to our emergency department or neighbouring minor injury units over a 2 year period were reviewed. We recorded demographics, mechanism of injury, direction of the dislocation radiologically, concomitant injuries and rates of follow-up (FU).

**Results:**

There were 74 dislocations in 74 adults with a median age of 46 (range 14–87) years. Fifty-six (76%) were men and 18 (24%) were women. The dislocations were ulnar more than radial: little finger (43%), ring finger (34%), middle finger (15%) and index finger (8%). Amongst all 74 dislocations, 36 (49%) were dorso-ulnar, 30 (41%) dorsal, five (7%) ulnar, two volar and one dorso-radial. Six dislocations were open. Five of these were dorsal dislocations and five affected the index (three) and middle (two) fingers. The median number of FU appointments was two (range 0–21).

**Conclusion:**

Our findings suggest that PIPJ dislocations commonly affect two distinct populations: young men suffering little finger dislocations during sport and older patients sustaining middle finger dislocations after falling. Open dislocations are predominantly dorsal dislocations of the index and middle fingers. FU rates are very variable but suggest 20% of cases have prolonged symptoms.

**Level of evidence:**

IV

## Introduction

Proximal interphalangeal joint (PIPJ) dislocations are common, up to 11.1 cases per 100,000 person-years (Golan et al., 2016), but their mechanisms of injury and outcomes have not been widely reported. They typically follow a sporting injury or a fall but can occur through a range of injury mechanisms (Golan et al., 2016; Joyce et al., 2014; Kolovich and Heifner, 2023). The limited published data gives a mixed picture.

Joyce et al. (2014) conducted a retrospective review of 77 PIPJ dislocations from the Republic of Ireland. Although the study focused on treatment options, the authors also documented the most commonly affected digits and noted that sporting injuries accounted for 71%. A national database epidemiological study in the USA again reported that sport was the most common mechanism of injury, comprising 70% of dislocations (Golan et al., 2016).

Kolovich and Heifner (2023) reported three types of PIPJ dislocations: dorsal, lateral and volar. However, this does not acknowledge the common dorso-lateral dislocation, which seems more frequent than a purely lateral dislocation. It is recognized that there can be a range of mechanisms of injury including falls (which fits with the upper limb falling reflex) (Giddins and Giddins, 2021) as well as catching injuries and others. The mechanisms of these injuries have not been reported in detail.

The aim of this study was to assess the mechanisms of PIPJ dislocations to provide a better understanding of the biomechanics of these injuries.

## Methods

We identified all PIPJ dislocations that presented to our hospital or local minor injuries units within a two-year period by reviewing every finger radiograph and associated radiology report taken in the Emergency Departments between 4 July 2022 and 3 July 2024. The inclusion criteria were skeletally mature patients (closed physes) presenting acutely (⩽7 days from injury) with a PIPJ dislocation, including both open and closed dislocations. The exclusion criteria were patients with open physes, old injuries and fracture-dislocations/subluxations as opposed to dislocations with small bony avulsions. We defined a fracture dislocation as a dislocation with a fracture involving at least 15% of the base of the middle phalanx on the postero-anterior or lateral radiographs.

Across the study period, 95 possible PIPJ dislocations were identified; 74 met the inclusion criteria and 21 were excluded. From the medical records we recorded demographics, date of presentation, mechanism of injury, hand and digit injured, the total number of follow-up (FU) appointments (virtual fracture clinic, fracture clinic and hand therapy) and whether the dislocation was open or closed. Both authors independently assessed all the radiographs recording the direction of dislocation and the presence of any bony injuries.

The Shapiro–Wilk test was employed to determine normality of continuous data, which revealed significant variation from a normal distribution (*W* = 0.94, *p* = 0.003). Fisher’s exact test was used to analyse differences between categorical data, while the Mann–Whitney *U*-test was applied to compare medians of non-parametric numerical data such as age. Statistical significance was defined as *p* < 0.05.

The hospital’s audit committee approved this study.

## Results

There were 74 PIPJ dislocations in 74 patients; 56 (76%) were men and 18 (24%) were women. The median age at presentation was 46 (range 14–87) years. ‘Sport’ and ‘fall’ were the most common recorded mechanisms of injury, accounting for 36 (49%) and 28 (38%) dislocations, respectively. The right hand was injured in 42 (57%) cases and the left in 32 (43%) cases; hand dominance was not recorded.

**Figure 1.**
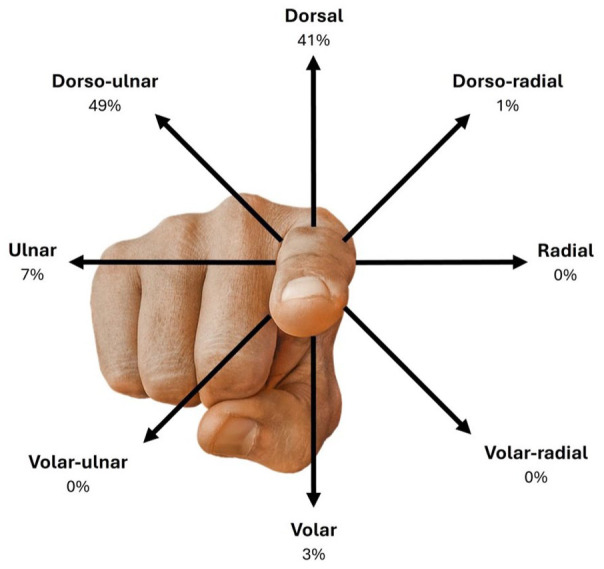
Relative incidence of dislocation direction across all fingers. Note that the percentages represent all the digits combined, not only the index finger.

**Figure 2.**
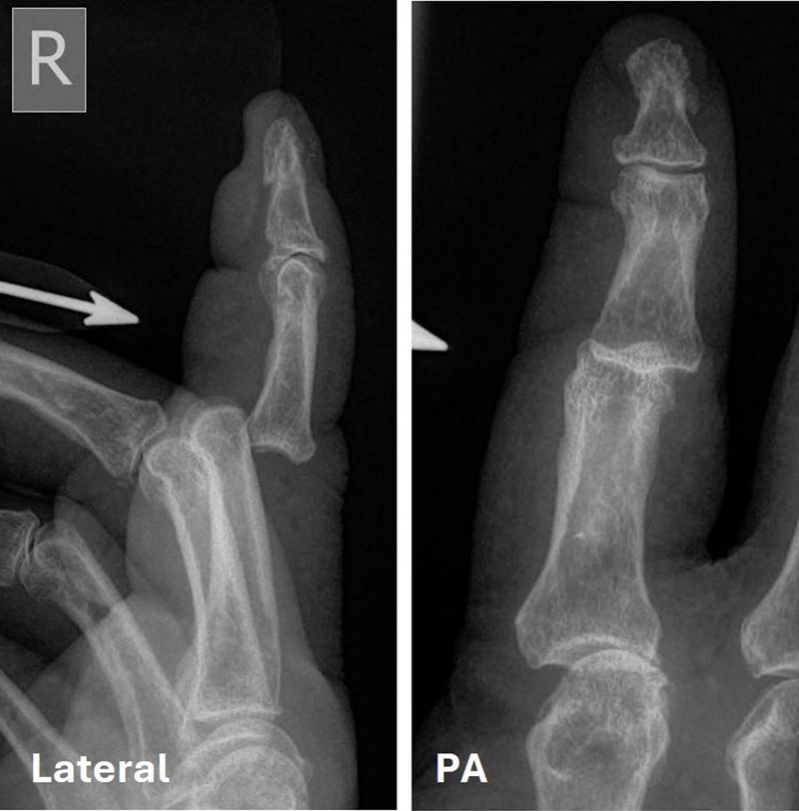
Radiograph showing an open dorsal dislocation of the index finger. Half (3/6) of all index finger dislocations were open.

The most frequently injured digit was the little finger (43%), followed by the ring finger (34%), the middle finger (15%) and the index finger (8%). The radiological direction of dislocation was dorso-ulnar in 36 (49%), dorsal in 30 (41%), ulnar in five (7%), volar in two (3%) and dorso-radial in one (1%) (Figure 1). There were no radial, volar-radial or volar-ulnar dislocations. Only six (8%) of the 74 dislocations were open, five of which were dorsal, affecting the index finger (three) and middle finger (two) (Figure 2). The sixth open dislocation involved the little finger and occurred in an ulnar direction; it was the only case that proved irreducible and required open reduction. The index finger sustained significantly more open dislocations than the ring finger (*p* = 0.0044) and the little finger (*p* = 0.0089). There were significantly more open dorsal dislocations compared with dorso-ulnar (*p* = 0.0159) across all digits.

A marginal bony avulsion injury was present in 40 of 74 (54%) dislocations. Patients with a bony avulsion injury were significantly older on average than those who did not (*p* = 0.0446 for a one-tailed Mann–Whitney *U* test; *p* = 0.0894 for two-tailed). Seven of 17 (41%) index and middle finger dislocations presented with an avulsion injury, compared with 33 of 57 (58%) ring and little finger dislocations (NS). Both volar dislocations resulted in a bony avulsion injury, one of the radial collateral ligament and the other of the volar plate.

**Figure 3.**
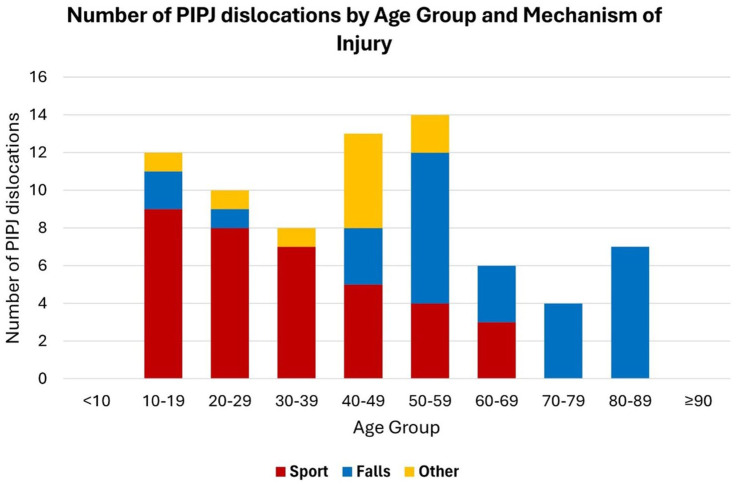
Number of proximal interphalangeal joint (PIPJ) dislocations by age group and mechanism of injury.

The mechanism of injury recorded in the medical records was often limited. Some of the sporting injuries may have been due to a fall. Figure 3 depicts the number of PIPJ dislocations, stratified by age and mechanism of injury. The number of dislocations caused by sport decreased until age 70 years, after which no patient suffered a sports-related injury. In contrast, the numbers owing to falls increased, especially among those over 50 years old.

The median number of FU appointments was two (range 0–21). When stratified by mechanism, injuries caused by sport or a fall both required a median of 1.5 FU appointments, whilst dislocations attributed to ‘other’ mechanisms had a median of 2.5 FU appointments. This category included assaults, a road traffic collision and incidents such as catching a finger in a dog collar, a whisk injury, or catching on a banister or in horse reins.

Based upon a catchment area for our emergency department of 500,000 there were around 7.4 PIPJ dislocations per 100,000 people per year, 11.2 per 100,000 in men and 3.6 per 100,000 in women (male–female ratio 3.1:1).

## Discussion

This study showed clear patterns of PIPJ dislocations that differ between the radial and ulnar sides of the hand. The most common were ulnar-sided injuries involving the ring and little fingers. These were typically closed, dorso-ulnar dislocations (the most common type) affecting a younger population. Radial-sided injuries (index and middle fingers) were less common and most often presented as dorsal dislocations. They were potentially more severe with half of all index finger dislocations being open.

These findings are comparable with two previous studies of similar cohorts, confirming that this is predominantly an injury of men that can occur at any age. Golan et al. (2016) reported 11.1 dislocations per 100,000 person-years (male–female ratio 3.8:1). Joyce et al. (2014) noted that the little finger was most commonly affected, followed by the ring, middle and index fingers. The older age group in our study is likely to reflect an older local demographic.

Our study adds to previous work in several ways. We have identified the direction of injury as predominantly dorso-ulnar most often affecting the little finger, often caused by sporting injuries and more commonly dorsal in the index and middle fingers, typically from a fall. In particular, we have identified the clear association of dorsal dislocations and open injures. Of the 30 dorsal dislocations, five were open, suggesting that there should be a higher level of suspicion when these patients are referred with limited medical information.

Volar, radial and irreducible dorsal dislocations are rare and tend to affect the little or ring fingers in an older population. Neither of the two volar dislocations occurred owing to a simple fall, but rather from a complex injury. This would fit with the upper limb falling reflex (Giddins and Giddins, 2021), with falling onto the outstretched hand leading to a dorsally directed force.

**Figure 4.**
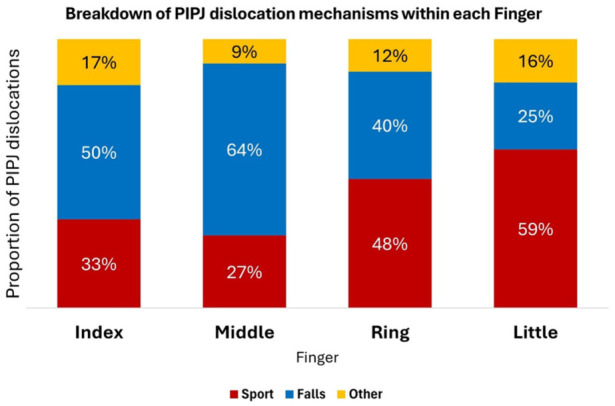
Proportion of proximal interphalangeal joint (PIPJ) dislocations by finger and mechanism of injury.

The reasons for the different directions of injuries are unclear. Mistimed and misdirected falls are likely to affect the longer digits, especially the middle finger, which would help explain the predominance of middle finger injuries after falls (Figure 4). The relative protection afforded by the ulnar digits may also reduce the chances of dorso-ulnar as opposed to dorsal injuries in the index and middle fingers.

None of the dorso-ulnar injuries were open despite it being the most common dislocation direction. Almost all open dislocations occurred in a dorsal direction and affected either the index or middle digits. Relative to the ulnar-sided digits, the index and middle fingers are stiffer at the carpometacarpal joints in conjunction with a more directly dorsal as opposed to a dorso-lateral force. This might lead to greater transmission of force through the PIP joints of these digits instead, leading to a higher incidence of open injuries.

Sports-related injuries most often affected the ulnar-sided digits. During sport, many injuries will be grabbing or oblique injuries. The ring and little fingers are likely to be more susceptible as they sit a little more palmar in the normal finger cascade and lack ulnar-sided support as afforded to the index and middle fingers.

The prevalence of bony avulsion injuries progressively increases from the index finger to the little finger while the proportion of open dislocations decreases. Even though older patients were more inclined towards index and middle finger dislocations (digits with a lower rate of bony avulsion injury), those with a bony avulsion injury were significantly older on average. This represents a predisposition towards avulsion injuries amongst the older population, probably related to relative tissue strength and stiffness (reduced bone density and ligament flexibility).

Joyce et al. (2014) reported a mean of 5.6 hospital visits per patient, whereas we found a mean of 2.8 (median 2). This inconsistency probably reflects variation in local practice rather than differing pathology. The treatment in our hospital was not standardized. Some patients were discharged from the emergency department with no FU whilst others were seen in the hand clinic or by hand therapy. Despite the inconsistency, patients with more FU appointments typically had greater symptoms recorded in the medical records. Around 20% of patients had appreciable short-term symptoms requiring more FU.

There are limitations to this study: in the hospital notes the distinction between ‘sport’ and ‘fall’ was not always clear. Several cases caused by falls might have occurred during sport but were registered as a sports-related injuries. Therefore, the number of dislocations caused by falls could be underestimated in the study. We may not have captured every PIPJ dislocation in the community if reduced outside hospital and the patient did not seek medical assistance. The clinic reviews were not standardized.

In conclusion, these data show a bimodal distribution of PIPJ dislocations, affecting young men during sport, and older patients sustaining middle finger dislocations after a fall. Twenty percent of patients appear not to do well initially.

## References

[bibr1-17531934251405707] Giddins G Giddins H . Wrist and hand postures when falling and description of the upper limb falling reflex. Injury. 2021, 52: 869–76. 10.1016/j.injury.2020.11.056 33358532

[bibr2-17531934251405707] Golan E Kang KK Culbertson M Choueka J . The epidemiology of finger dislocations presenting for emergency care within the United States. Hand (NY). 2016, 11: 192–6. 10.1177/1558944715627232 PMC4920528 27390562

[bibr3-17531934251405707] Joyce KM Joyce CW Conroy F Chan J Buckley E Carroll SM . Proximal interphalangeal joint dislocations and treatment: an evolutionary process. Arch Plast Surg. 2014, 41: 394–7. 10.5999/aps.2014.41.4.394 PMC4113700 25075363

[bibr4-17531934251405707] Kolovich GP Heifner JJ . Proximal interphalangeal joint dislocations and fracture-dislocations. J Hand Surg Eur. 2023, 48: 27S–34S. 10.1177/17531934231183259 37704028

